# Development of mental healthcare in Cambodia: barriers and opportunities

**DOI:** 10.1186/s13033-020-00385-4

**Published:** 2020-07-29

**Authors:** Sarah J. Parry, Nil Ean, Shirley P. Sinclair, Ewan Wilkinson

**Affiliations:** 1 OMF International (Cambodia), #3, Street 604, Tuol Kork, PO Box 570, Phnom Penh, Cambodia; 2 Department of Psychology, Royal University of Phnom Penh, #110, Russian Federation Boulevard, Phnom Penh, Cambodia; 3 Institute of Medicine, University of Chester, Parkgate Road, Chester, CH1 4BJ UK

**Keywords:** Mental healthcare system, Training, Quality, Collaboration, Sustainable development, Culturally appropriate, Qualitative

## Abstract

**Background:**

Despite the increasing recognition globally of the importance of mental health for sustainable development, significant barriers remain to developing mental health services in low- and middle-income countries. This study explored the particular barriers and opportunities for developing mental health services in Cambodia and how these compared with those described in other low- and middle-income countries.

**Methods:**

For this qualitative study, 18 experienced mental health professionals from different disciplines were selected using purposive sampling. Semi-structured interviews were carried out in Phnom Penh and thematic analysis of the data was completed.

**Results:**

Five key themes were identified: (1) Prioritising mental health in Cambodia, (2) Strengthening collaborations between mental health stakeholders, (3) Developing a mental healthcare model appropriate for the Cambodian culture and context, (4) Increasing the quantity and (5) Improving the quality of mental healthcare. All five themes were referred to by all 18 participants and the two most repeated themes were (2) Strengthening collaborations and (5) Improving the quality of mental healthcare.

**Conclusions:**

The themes identified in this study both corroborate previous barriers identified to developing mental health services in low- and middle-income countries and shed new light on opportunities of particular importance in Cambodia. Strengthening collaborations between key stakeholders in mental health and prioritising the quality of mental health education, training and service provision were both cited as being significant opportunities for enhancing the development of mental health services in Cambodia. These have not been widely described before as being important factors.

## Background

The importance of addressing the global burden of mental disorders has been increasingly recognised in the last decade. The first “Lancet” Series of articles on global mental health in 2007 called for urgent action to scale up mental health services in low- and middle-income countries (LMIC) [1]. This was followed by the launch of the “*Mental Health Gap Action Programme”* by WHO in 2008 [2]. The inclusion of mental health indicators in the Sustainable Development Goals in 2015 represented an important step in raising the global priority of mental health [3, 4]. The Lancet Commission on “Global mental health and sustainable development” in 2018 again highlighted the ongoing challenge of reducing the global burden of disease attributable to mental disorders in LMIC [4].

Despite these reports, resources remain scarce for mental health services in many LMIC [4–6]. There remains a treatment gap (the difference between the number of people with mental disorders who need care and those who receive care) in LMIC, as well as a significant prevention gap (the gap in the coverage of interventions focused on targeting mental health risk factors) and a quality gap (the mismatch between the quality of care that should be delivered for people with mental disorders and the quality of care that is delivered) [4].

Progress in developing mental healthcare in LMIC continues to meet significant barriers and challenges. Barriers include: the low priority of mental health in comparison to other public health agendas [5–10], lack of political will [6–8, 10], inadequate funding and resources for mental health services [5–10], the challenge of decentralisation and integrating mental health services into a community setting [6–8], the shortage of public health trained mental health leaders [6–8] and the absence of service user involvement in development of mental healthcare [9].

These barriers were reported in studies in LMIC worldwide. There have been no studies focused on the barriers to developing mental health services in Cambodia, a country which has faced significant socio-economic challenges in recent years [11–13].

Cambodia is a country in Southeast Asia with a population of 15.3 million [14], bordered by Vietnam, Thailand and Laos. Cambodia ranks 146 of 189 countries in the United Nations Human Development Index [15].

The majority (97%) of the population are Buddhist [19], however the concept of health in Cambodia is also influenced by Hinduism, animism and concepts of luck and astrology [20]. Healthcare is often sought in a pluralistic fashion through a combination of self-medication, traditional healers or “Kru Khmer”, monks, the public health system, non-governmental organisations and private healthcare facilities including pharmacies and clinics [16, 20, 21].

Hierarchy has a high value in Cambodian culture; in as early as the thirteenth century legends about Angkor illustrate the strictly hierarchical culture and the importance of patron-client principles [16]. Social and professional relationships today in Cambodia continue to be shaped by hierarchy and patronage [16, 17]. Patron-client relationships, based on mutual obligation, also arise within state operations and public service provision [18]. Relationships may be damaged if reciprocity is neglected and so organisational change may happen more gradually, however valuable relationships may be preserved.

During the Khmer Rouge rule and preceding civil war in the 1970s, the Cambodian people experienced a prolonged period of violence and brutality, leading to the death of a third of the population [22]. After the 1991 Paris Peace Agreement was signed, a “liberal, constitutional democracy” [17] was introduced and since 1991 the Cambodian People’s Party has remained in power. 

The development of mental health services in Cambodia has been greatly influenced by the destruction of all infrastructure and health services during the Khmer Rouge period until early 1990s [19, 23–25]. Prior to the conflict, there was a single 800 bed psychiatric hospital with a patient population of approximately 2000 [12, 13]. All of the mental health professionals and patients were killed during the Khmer Rouge period and the hospital has not been re-opened [11, 13, 21, 24].

Consequently, Cambodia did not have the challenge of de-institutionalisation of mental healthcare unlike many of its neighbouring countries in Southeast Asia [13, 24]. This presented an opportunity for services to be developed in a decentralised manner [12, 13]. However, Cambodia has been presented with the bigger challenge of rebuilding a mental health service in its entirety alongside the rebuilding of the country’s infrastructure and economy [11, 13, 23, 26]. Early international partnerships and collaborations with NGOs had a significant influence on the initial rebuilding of mental healthcare [23, 25]. Trust in NGOs continues to be high and they are permitted an active role by the government if they remain deferential and apolitical [18].

In 1992 the Cambodian Ministry of Health formed the “*Mental Health Subcommittee*” to develop mental health services [12, 13]. In 1994 the department of psychology at Royal University of Phnom Penh (RUPP) was established [20] and short courses in counselling became available through NGOs [27]. From 1994, 26 psychiatrists, 40–45 psychiatric nurses and 600 primary care doctors and nurses in basic mental healthcare were trained through international partnerships [11, 12, 24, 28]. After international funding ended in 2006, the training opportunities were reduced [11, 19, 23]. There are currently 60 psychiatrists in Cambodia [19] and the University of Health Sciences offers a 3 year psychiatry residency program for doctors [12]. In 2008, the social work department at RUPP was established and the department of psychology launched a masters program in clinical psychology [20]. However, the psychiatric nurse training program has not restarted since it ceased in 2006 [11, 12] and the number of primary care professionals with basic mental health training remains at 600 [22].

Funds and resources for mental healthcare in Cambodia are limited, especially in rural areas [19]. The total number of psychiatric inpatient beds has remained at 10–15 since 2010 [11, 12, 19, 26, 28]. There is inadequate access to psychotropic medications and no mental health legislation [19]. There have been several attempts to create and implement a mental health policy and plan [11, 13, 24], however these are yet to come to fruition [19]. The “*2016–2020 Health Strategic Plan”* by the Ministry of Health makes specific mention of the need to develop the services for mental health disorders and substance abuse in Cambodia and acknowledges the current situation requires attention [29].

In light of Cambodia’s history, mental health research and the work of non-governmental organisations has been largely focused on trauma related care [23, 30]. More recently there has been a recognition of the need for a focus on common mental disorders and a number of prevalence studies have been carried out in Cambodia. Epidemiological data consistently shows high rates of anxiety, depression and post-traumatic stress disorder (PTSD) in Cambodia. A recent cross-sectional study showed rates of depression as 16.7%, anxiety as 27.4% and PTSD 7.6% [31]. Previous studies have shown the prevalence of depression ranging from 11.5 to 80%, anxiety as high as 53% [20, 31–33] and PTSD ranging from 7 to 86% [20, 23, 31–34]. The prevalence of suicide has been estimated at 13–44 per 100,000 [19, 20, 35]. The prevalence of substance misuse disorders has been estimated as over 2% [36], with the main drugs used in Cambodia being amphetamine type stimulants, cannabis, heroin and opium [23, 36, 37]. A significant rise in methamphetamine use has been noted and the population prevalence of methamphetamine use was estimated as 4% in 2001 [38]. There remains a need for large scale prevalence studies for substance use disorders [38] and severe and enduring mental disorders in Cambodia [39].

Studies focusing on child and adolescent mental health in Cambodia have shown high rates of depressive symptoms in young people [40] and high exposure to trauma [41]. The prevalence of epilepsy in a cross-sectional study was found to be 5.8 per 1000 [42]. There remains a need for further prevalence studies of mental, neurological and behavioural disorders of children and adolescents in Cambodia [23].

In Cambodia, significant work has taken place to describe Cambodian culture-bound syndromes and idioms of distress, particularly relating to trauma in both adults [43–45] and children [41, 46]. Examples described in Cambodia include “baksbat” (broken courage) [47] “kut chraen” (thinking too much) [21, 41, 47, 48] “mour mao” (easily angered) in children [41] and “khyal” (windlike substance) attacks (in Cambodian refugees) [49]. The importance of contextualised mental health research has been highlighted as crucial to developing accurate diagnostic criteria, effective interventions and appropriate long-term follow up [43, 45, 47, 50]. In light of the significant challenges that the development of mental health services in Cambodia has faced, this study was conducted to explore the barriers and opportunities for furthering the development of mental health services in Cambodia.

## Methods

Purposive sampling was used by the authors EN, SS and SP to select a balance of highly experienced professionals from different disciplines and working environments. A sample of 18 mental health professionals based in Phnom Penh participated in this study. Invitations were sent by EN and SP to a total of 20 potential participants, of whom 2 did not respond. Participants were not known personally to the interviewer SP. The invitations explained the purpose and format of the study: to learn from highly experienced mental health professionals about the barriers and opportunities of developing mental healthcare in Cambodia. Participants were given an information sheet and signed an informed consent form prior to participating. 

Semi-structured interviews were conducted using seven questions adapted from the questionnaire used in a previous WHO study [7, 8] but focusing on Cambodia. Questions were adapted for the Cambodian context and culture and translated into Khmer by EN. Copies of the questions in both Khmer and English were given to each participant at the start of the interview. The questions used in the interviews are available in the appendix (Additional file 1 and 2).

Fifteen of the interviews were carried out in English and three of the interviews were in English and Khmer with an interpreter, depending on the participants’ choice. The interviews in English were carried out by SP, and the interviews in Khmer by SP with a Cambodian postgraduate student in counselling with a high level of English and previous experience of employment as an interpreter. Each interview was recorded and transcribed verbatim by SP. A second interpreter checked the interpretation using the audio recordings of the interviews in Khmer.

The transcriptions totalled over 100,000 words and were inputted into NVivo [51] and thematic analysis [52] was completed by the authors. An essential, inductive method of thematic analysis was used according to the step-by-step methods in Braun & Clarke (2006): *“Using thematic analysis in psychology” *[52]. Themes were identified at a semantic level in order to describe the entire data set [52]. The key themes were developed by SP and refined, edited and agreed on by all four authors.

## Results

Demographics of the participants are detailed in Table 1. The three non-Cambodian participants had several years of experience living and working in Cambodia.

**Table 1:** Demographics of participants interviewed for this study in Phnom Penh, 2019

Demographics of participants	No. of participants
Gender	
Male	10
Female	8
Nationality	
Cambodian	15
Non-Cambodian	3
Professional discipline	
Psychiatrist	6
Psychologist	4
Social worker	3
Counsellor	2
Psychiatric nurse	2
Government officer	1
Primary work setting	
Government	11
NGO/private	7

**Fig. 1:**
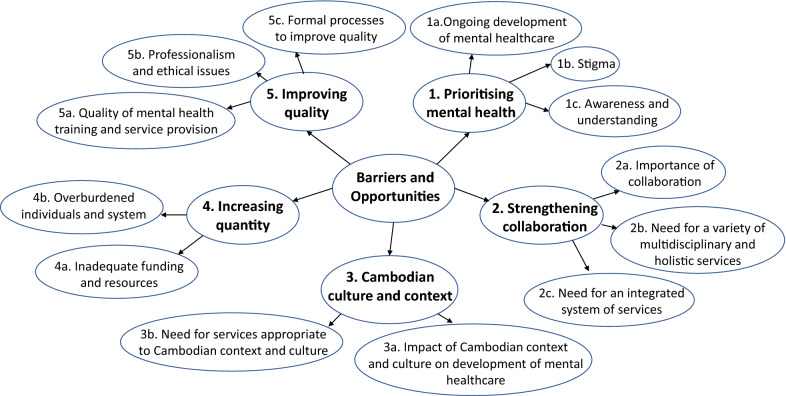
Key themes and subthemes identified from 18 interviews of mental health professionals in Cambodia, 2019

Five key themes were established through the process of thematic analysis (see Fig. 1).

The total number of times each theme was mentioned is detailed in Table 2 along with the total number of participants who made references to each theme. The subthemes within each key theme are displayed in Table 3 along with the number of times participants referred to the subtheme, and the total number of participants who referred to each subtheme.

**Table 2:** Number of mentions of each theme by participants during interviews on development of mental healthcare

Key theme	Total no. of references	No. of participants
1. Prioritising mental health in Cambodia	443	18
2. Strengthening collaborations between mental health stakeholders	592	18
3. Developing a mental healthcare model appropriate for the Cambodian culture and context	387	18
4. Increasing the quantity of mental healthcare	289	18
5. Improving the quality of mental healthcare	508	18

**Table 3:** Number of participants referring to each subtheme and total number of references to each subtheme

Key theme	Subtheme	No. references	No. participants
1. Prioritising mental health in Cambodia	1a. Ongoing development of mental healthcare	239	18
	1b. Stigma	29	13
	1c. Awareness and understanding of mental health	175	18
2. Strengthening collaborations between mental health stakeholders	2a. Importance of collaboration	306	18
	2b. Need for a variety of multidisciplinary and holistic services	169	17
	2c. Need for an integrated system of services	117	17
3. Developing a mental healthcare model appropriate for the Cambodian culture and context	3a. Impact of Cambodian context and culture on development of mental healthcare	283	18
	3b. Need for a service appropriate to the Cambodian context and culture	104	17
4. Increasing the quantity of mental healthcare	4a. Inadequate funding and resources	221	18
	4b. Overburdened individuals and system	68	16
5. Improving the quality of mental healthcare	5a. Quality of mental health training and service provision	326	18
	5b. Professionalism and ethical issues	88	13
	5c. Formal processes to improve quality	94	15

Looking at the key themes in more detail:

## Developing mental healthcare together

### 1. Prioritising mental health in Cambodia

#### 1a. Ongoing development of mental healthcare in Cambodia

There has been significant development of the mental health services in Cambodia in recent years. The growth in education, training and resources for mental health was highlighted by sixteen participants:

“I started from zero so I experienced all opportunity for mental healthcare development. From having nothing, we have create[d] now sixty psychiatrists and there are about seventeen psychiatric residents who are still in the training. We train[ed] forty psychiatric nurses”.

Despite this notable progress in training professionals, there was also a sense of frustration among participants concerning the development of mental health services in Cambodia compared to other areas of development and recognition that more progress could have been made:

“Given that it started  twenty plus years ago; the development; it hasn’t gone anywhere near far enough I mean twenty years ago people would say ‘Oh well we have to think about the economic development first, you know, enough food to eat’, that sort of thing. And that’s gotten better, but there hasn’t been an accompanying development of mental health.”

Participants highlighted that in order to effectively raise the priority of developing mental health services, the development of a national mental health strategy and mental health legislation are necessary. Beyond simply developing a strategy, there were also calls for proper implementation of policy:

“Lack of policy, [on a] global level, [and] the country level. Mental health policy document[s] [in Cambodia], were done twice: one with the help of WHO, another PRIME, so policy [is] on paper, but when it comes to implementation there is a big gap.”

#### 1b. Stigma

Participants highlighted the significant stigma and discrimination associated with mental disorders in Cambodia:

“Because the term mental health in Cambodian, when we translate [it], it means you are crazy. So if you [are] crazy you are discriminate[d] against […] people [are] afraid to be infected by this disease.”

#### 1c. Awareness and understanding of mental health

All participants commented on issues relating to raising awareness of mental health. Participants highlighted the importance of empowering people with knowledge of the causes and available treatments for mental disorders, to enable people to seek appropriate care and support:

“With more information, with more knowledge, people have more choice. So by empowering people to have more choice about their mental health and their mental healthcare, including how they can take care of their mental health I mean, I think it’s the most important thing.”

The importance of raising awareness of mental health amongst those in positions of leadership was particularly emphasised:

“And [we need to] reinforce capacity building within the ministry as well, not only staff, not only psychiatrist, not only nurse, but also the leader; to really understand what is mental health and why it’s very important.”

Tools that were mentioned as being particularly useful for raising public awareness and knowledge in Cambodia included TV, radio and social media (Table 4):

“With 16 million people [in Cambodia], there are 5 million to 6 million people using Facebook. So I think […] giving education through social media is very helpful.”

**Table 4:** Summary of barriers and opportunities within theme 1: Prioritising mental health in Cambodia

Barriers
Low priority of mental health compared to other development priorities
Incomplete implementation of mental health policy
Stigma and discrimination against people with mental disorders
Lack of awareness and understanding of mental health amongst leaders and the general population
Opportunities
Development and effective implementation of mental health policy and legislation
Sustained efforts to raise awareness, especially amongst leaders
Utilising modern technology e.g. social media for education, to raise awareness and reduce stigma

### 2. Strengthening collaborations between mental health stakeholders

#### 2a. Importance of collaboration

Participants noted that individuals and organisations working in mental health in Cambodia often work independently rather than together and the services themselves lack an integrated system for referral. The lack of and need for collaboration, including between and within different professional disciplines as well as between individuals, organisations and departments, was commented on by every participant:

“The government and the non [government] organisation, they work like individually. Same area, same area, I mean same issue […] they work alone, you know, they work alone.”

Reasons suggested for the lack of collaboration included a lack of trust, personal conflicts, differences in priorities between individuals and organisations and issues around status and professionalism:

“A long period of civil war and a long history of trauma among our people might also contribute to the lack of trust among our people.”

“Well, professional silos, the: ‘We did this best so, you know, now we don’t have to talk to other people’. Or you know, ‘They’re only psychologists or social workers and we’re doctors and we know better’, that whole back and forth.”

Interestingly, every participant also emphasised that strengthening collaborations could be beneficial to improving the mental health services. The benefits of collaboration for sharing knowledge in education and training between and across disciplines was highlighted:

“One way is meeting, training and sharing knowledge, sharing idea, sharing opinion to each other (…) When we find anything new, so [if] we share that to people, to some organisation that work in that area also, [this] is one way to improve each other.”

Furthermore, it was suggested that meeting together for planning and developing strategy could be beneficial:

“I think something like the [previous] mental health subcommittee could be an incredibly useful tool.”

#### 2b. Need for a variety of multidisciplinary and holistic services

Participants also suggested that greater collaboration in service provision could also enable the creation of a multidisciplinary team, as currently professionals from different disciplines tend not to work together:

“But now you know in Cambodia, it still a problem, psychiatrists and psychologist [do] not work together.”

A multidisciplinary team would enable services to offer a more holistic model of care:

“Because now like Ministry of Health they try to have mental health service […] I think they should include psychologists and social worker to work in these services, I think its [would be] good, because now [there are] only psychiatrists. I think if they include three together to work with the mental health service in the hospital, I think it [would be] good.”

#### 2c. Need for an integrated system of services

A more integrated system of services would also enable greater coordination of care across the village, district and provincial level:

“The challenge was to integrate the rest of the health system. That has not happened so far […] They [the mental health services] are not fully integrated with the three-tier healthcare system of primary, secondary, tertiary. So that’s a challenge.”

Several participants mentioned successful collaborations with international partners yet very few gave examples of successful collaborations between Cambodian individuals and organisations (Table 5).

**Table 5:** Summary of barriers and opportunities within theme 2: Strengthening collaborations between mental health stakeholders

Barriers
Lack of collaboration between professional disciplines
Few collaborations between NGOs, government services, institutions and departments
No integrated system of referral between services
Opportunities
Meeting of key stakeholders to develop a vision and action plan, strategy and developing policy
Organising multidisciplinary training sessions to facilitate sharing knowledge
Working to develop relationships between mental health professionals
Encouraging the development of multidisciplinary mental healthcare teams and referral pathways between services

### 3. Developing a mental healthcare model appropriate for Cambodian culture and context

#### 3a. Impact of context and culture on the development of mental healthcare

The post-conflict context and stage of economic development has shaped how mental health services have developed in Cambodia over the last 30 years [19, 23, 24]. These factors continue to have a significant impact on both the mental health of the population in Cambodia, as well as the resources and facilities available for providing mental healthcare:

“From 1979 until now, millions of people who suffered, millions of people who were survivors and their children, meaning the new generations, even [though] they never experienced Pol Pot time but they experience post war times, they also experience psychiatric disorder, […] they could not get access to mental healthcare, because of realistic [availability of] facilit[ies] in the country.”

Participants also expressed how aspects of Cambodian culture have influenced the development of mental healthcare. For example, in Cambodia there is a high value placed on authority and hierarchical structures [47, 53, 54] and therefore an expectation for leadership to take responsibility:

“I think because the Ministry of Health sort of claims that role of leadership, there isn’t a lot of external leadership you know. People don’t feel like they have the right to exert leadership I guess.”

The importance of respecting authority was said to lead to a reluctance for people to voice their opinions:

“So [if] somebody stands up and says: […]‘This is what you should do’; no one wants, no one dares to do that typically [stand up and speak out].”

There was a sense that participants did not feel empowered to be agents of change if they were not in a position of authority:

“After all what you are talking with me, its just what you are talking with me, I am not a decision maker.”

#### 3b. Need for a service appropriate for the Cambodian culture and context

Participants discussed the need for culturally appropriate services, taking into consideration health beliefs prevalent in Cambodia:

“Because people in the country believe that acute clinical manifestation of any psychiatric disorder […] is linked to the spirits. So no one can heal with their [illness], only the traditional healer and the monks.”

Participants highlighted the need to adapt models of care to the Cambodian context, rather than importing purely Western models of mental healthcare.

“To apply the Western style of counselling sometimes might not really [be] helpful, we have to adapt all of those things in order to [provide a] more effective intervention. I can say for example when we [are] talking about [psychological] testing, most of the tests are not valid in Cambodia.”

Several participants commented that although most of the population live in rural areas [19, 23, 55], most services are currently offered in urban areas, primarily Phnom Penh [19]. The need for investment in community services in rural areas was emphasised, which would also be appropriate, given the strength of community values in Cambodia:

“So that support system is very strong in countries like Cambodia, and Asia, where the family network is good. You know, so it’s important to strengthen the existing systems of care in the community.”

Participants suggested that focusing on developing basic mental healthcare at the community level would be most appropriate for the current Cambodian context (Table 6).

“I think that the way to develop mental health services has to be community based mental health, because there will never be enough resources for mental health in Cambodia [in terms of] professionals”

**Table 6:** Summary of barriers and opportunities within theme 3: Developing a mental healthcare model appropriate for Cambodia

Barriers
Societal impact of post-conflict context
Western models of mental healthcare imported without cultural adaptation often less effective
People who are not in positions of formal leadership may be reluctant to offer opinion due to cultural expectations
Services concentrated in urban areas inappropriate for rural context
Opportunities
Developing further culturally appropriate models of mental healthcare from the existing base
Focusing on developing appropriate and accessible community services

## Raising the level of service

### 4. Increasing the quantity of mental healthcare

#### 4a. Lack of funding and resources

Inadequate funding and resources were repeatedly mentioned as barriers to developing mental health services in Cambodia. The government funding for mental health as well as the external, international funding were both said to be inadequate:

“According to what I know from the government they spend less than 1% of the annual budget for mental health. Less than 1% […] they have around 100,000 US dollar to spend on mental health [per year], for the whole country.”

“But unfortunately our project finished last year because the fund from […] ends, and actually there is no funding at all for mental health in Cambodia, especially for community mental health. Our funding mainly come[s] from the […] umbrella of gender based violence, human rights, tortures, trafficking.”

As well as funding being reported as inadequate, it was also frequently mentioned that sources of funding were often stopped or reduced, leading to some resources decreasing over time rather than developing. One example of this was that the psychiatric nurse training was discontinued in 2006 [11] and has not restarted.

“There is no more training of psychiatric nurse, […] there will be no more person who will study as psychiatric nurse.”

The fluctuations in donor funding for mental health was also highlighted as problematic:

“Some NGO they, their fund is being deducted because it really depend[s] on external donor[s].”

“If the [non-government] organisation do[es] it, [then] just only gone, and [at one point you] have and [then its] gone. Like example, if one day like if the hospital also cannot take it over, cannot pay, and then if the [non-government funded] project gone, no money anymore and then its gone.”

“Because NGO only come and go, and NGO cannot cover the whole country”

Inadequate resources for mental healthcare were frequently mentioned, including human resources and services, a lack of medications and inadequate buildings. The lack of mental health services in rural areas was emphasised, as was the lack of inpatient facilities for mental health.

“So there is a shortage of mental health professionals, including psychiatric nurses, social worker, counsellor, clinical psychologist, psychotherapist, psychiatrist. And we don’t have this network, we don’t have this human resource”

“There is no financial support [for mental health services], there is no facility, we have only the outpatient, no inpatient, except in Phnom Penh.”

#### 4b. The existing overburdened system

Many participants highlighted that the inadequate level of human resources and funding leads to the existing services being overwhelmed:

“So many client will come to the hospital where we have service. So its crowded. You know like in here […], we have just about 10 psychiatrist working there, but they need to deal, provide service for about 500 client per day.”

As well as the services themselves being overwhelmed, participants reported that individuals working in mental health often have several roles in different programmes and the attrition rate is high (Table 7):

“[At the] health centre level, one staff may need to work with the mental health, with the substance abuse, with other national program like immunisation.”

“One of the challenges of the workforce is that many people trained in mental health in Cambodia, many of them quit, they burnt [out], they go into other health related fields.”

**Table 7:** Summary of barriers and opportunities within theme 4: Increasing the quantity of mental healthcare

Barriers
Inadequate funding and resources
Fluctuating donor funding
Attrition of mental health professionals
Opportunities
Advocating for mental health funding
Increasing staff support and pastoral care to prevent attrition and burnout

### 5. Improving the quality of mental healthcare

#### 5a. Quality of mental health training and service provision

In addition to increasing the quantity of funding and developing resources for mental health in Cambodia, a key issue discussed by every participant was the importance of quality. There were several areas of concern that participants discussed. These included the quality of education at school, university and in professional training:

“Even school education is […] very poor. You may know [in] the whole of Asia, the lowest school hours is [in] Cambodia, only four hours per day. You know, so because schooling is poor the same reflects at the university level.”

The poor quality of mental health professionals, leaders and service provision was also highlighted repeatedly:

“They judge themselves: ‘I am a counsellor because I get two week[s] training on basic counselling’, […] in Cambodia […] anyone they can say that ‘I am a counsellor’.”

“What I see [regarding] the gap about the medical profession, physician who got training, they did not get training on interpersonal communication skill, they are very aggressive and use bad language also with patient.”

“We have lack of a good leader […]; the leaders that can coordinate, embrace everyone; but we lack of that.”

#### 5b. Professionalism and ethical issues

Related to quality, thirteen participants raised concerns regarding professionalism and ethical issues:

“For example: I am a doctor, I am a psychiatrist, and you come to meet with me; I can understand that your situation [is] not really serious, so you might need counselling rather than medication. But I attempt to provide you medication rather than refer you for counselling or psychotherapy because if I refer you to [a] counsellor I will [have] lost my benefit […] It like we [are] feeding a pet but we will not give them enough food, to make them come back to meet with us again.”

“In the report [to senior management] it seem that there are a lot [of good results] but practically it is nothing. Because Cambodia is the country of report. They report everything very pretty, but in practice nothing change.”

#### 5c. Formal processes to improve quality

In light of the concerns regarding quality and professionalism, several formal processes and actions were suggested by participants to raise standards of mental health services. These included developing professional guidelines, introducing licensing and accreditation for practitioners, creating professional associations, undertaking regular monitoring and evaluation of services and providing clinical supervision and support for professionals.

“Until now we don’t have any standard of practice, ethical framework, we don’t have that thing. We don’t have. And also we do not have any accreditation from any institute or from any government sector to accredit that we are qualified to do the job [counselling].”

“There should be an association, that give[s] credentials or control[s] or supervise[s] the practice of psychiatry or give[s] knowledge, continue education and so on.”

There was a clear sense from participants that these formal processes could contribute to improving the quality and effectiveness of mental health services in Cambodia. In addition to increasing the availability of services, there was a strong desire to prioritise raising the quality and standards of mental healthcare (Table 8).

**Table 8:** Summary of barriers and opportunities within theme 5: Improving the quality of mental healthcare

Barriers
Poor quality of education at all levels, service provision and leadership
Lack of professionalism and ethical framework in mental healthcare
Opportunities
Investment in mental health education at all levels
Development of professional guidelines and ethical frameworks for psychiatrists, psychologists, counsellors and social workers
Development of professional associations for mental health practitioners
Licensing and accreditation for mental health practitioners by professional body
Regular monitoring and evaluation of mental health services
Investing in clinical supervision and support for mental health professionals

## Discussion

This is the first study to explore the barriers and opportunities to developing mental health services in Cambodia and how they compare with those previously identified in other LMIC [7]. It is one of few studies to identify the need to improve the quality of the services at least as much as the quantity of services and that improving collaboration between key stakeholders could enhance the effectiveness of mental healthcare.

The first key theme, “Prioritising mental health”, echoes the recognition that mental health often has a low priority in public health agendas in many countries [5–7, 10]. Raising awareness both at the level of the general public and within those in leadership positions is key for raising the priority of mental healthcare in Cambodia and reducing stigma. Opportunities to challenge the current slow progress of developing mental health services include prioritising developing and implementing policy and investing in raising awareness and improving understanding through utilising modern technology.

The importance of culturally appropriate mental healthcare has also been recognised previously [56–59], resonating with the third theme, “Developing a model for the Cambodian culture and context.” Mental health service development has to be affordable and appropriate for the current situation in Cambodia. The post-conflict societal context, cultural influences and health beliefs in Cambodia require additional efforts to develop community-based mental health services that are appropriate for the country’s current needs.

The fourth theme, “Increasing the quantity”, reiterates the well documented barrier of inadequate funding and resources for mental health in LMIC and elsewhere [5–8]. Despite the increase in resources for mental health in Cambodia in recent years, the current funding and resources are inadequate and the overwhelmed workforce and system are struggling to cope with the demands of service users.

The two remaining themes identified in this study: “Strengthening collaboration” and “Improving the quality”, have only been identified in a small number of previous studies. Yet interestingly, these two themes were mentioned most frequently by participants (see Table 2).

Strengthening collaborations between key individuals, organisations and departments working in mental health in Cambodia was seen by participants as an important approach to enhance mental health service development. Although the importance of collaboration for successful mental health development has been recognised in some previous studies [60, 61], our study suggests lack of collaboration is a particular issue in Cambodia. The strength with which the participants spoke about the subject of collaboration gave a clear message; building collaborations between key stakeholders involved in mental health in Cambodia is crucial. The question of how to build sustained collaboration between individuals and organisations working in mental health is one that requires further investigation. Topics for future research could include exploring the methods and benefits of achieving collaboration between key stakeholders in mental health development.

The number of times participants referred to the need to improve the quality of mental healthcare and the effectiveness of services, beyond merely the availability of resources, was striking. The call from mental health professionals in Cambodia was clearly to prioritising quality rather than simply increasing the quantity of available resources. This is a reminder that even if there is greater public awareness of mental health and greater financial provision for developing services, without a high level of quality, mental healthcare will not be effective. There is a need to address the “quality gap” and not just the “quantity gap”[4]. Implementing and evaluating the value of proposed formal processes suggested by participants would be a valuable area for further research.

## Limitations

There were some limitations of this study. Fifteen of the eighteen interviews took place in English despite not being the first language of the participants but the level of English language proficiency among participants was very high. English is also a common language used in professional training in Cambodia, for example the psychiatry residency training is in English.

All of the interviews were carried out by SP, rather than a Cambodian professional. This was a pragmatic decision as SP was working on a voluntary basis and there was no budget to pay local professionals to carry out the interviews. It was also considered that due to the sensitive nature of some of the interviews, interviewees may feel more comfortable to speak with a neutral “outsider”, rather than someone working within the context. Participants may not have felt able to share their views in their entirety despite assurances of anonymity and confidentiality, due to the high value placed on hierarchy and the dynamic of patron-client relationships in Cambodia.

Views were only gathered from experienced mental health professionals based in Phnom Penh. Although most mental health professionals work in Phnom Penh [22, 23], the views of those working in rural areas would also be valuable. Finally, this research focused only on the professional view and did not involve service users.

## Conclusion

This study indicates two important opportunities for developing mental health services in Cambodia: Firstly, strengthening collaboration between those involved in mental healthcare on every level in order to build an integrated, holistic, multidisciplinary mental health service. Secondly, implementing formal processes to improve the quality of mental health education, training and service provision. This study also reiterates the importance of other well recognised challenges to developing mental healthcare in LMIC: prioritising raising the profile of mental health, developing a contextualised model for mental health services and increasing the quantity of funding and resources for mental healthcare.

Future research to explore the development of mental health services in Cambodia should consider the implementation of initiatives to improve quality and collaboration. Further evaluation of the effective methods to scale up of mental health services continues to be needed in Cambodia.

## Supplementary information

**Additional file 1.** Questions used in interview (English).

**Additional file 2.** Questions used in interview (Khmer).

## Data Availability

The datasets generated and/or analysed during the current study are not publicly available due to the risk of compromising the individuals who participated in this study. 9 participants agreed to be acknowledged and 9 participants requested not to be acknowledged for participating in the study. As the content of any of the interviews may reveal the identity of participants, in order to protect the identity of the participants who requested not to be acknowledged, we have therefore not acknowledged any of the participants and the data set is not publicly available. However, the corresponding author will consider reasonable requests.

## Abbreviations

LMIC: Low and middle-income country
NGO: Non-governmental organisation
RUPP: Royal University of Phnom Penh
WHO: World Health Organisation
PTSD: Post traumatic stress disorder
