# Supplementary material for: Development of mental healthcare in Cambodia: barriers and opportunities
Source: Int J Ment Health Syst. 2020 Jul 29;14:53. doi: 10.1186/s13033-020-00385-4 (PMC7392648; doi:10.1186/s13033-020-00385-4)
Supplement: Supplementary file 1 — Additional file 1. Questions used in interview (English). [file 13033_2020_385_MOESM1_ESM.pdf]

OMF International
#3, Street 604
Tuol Kork
Phnom Penh
Web: www.omf.org

Study Title: Development of mental healthcare in Cambodia: barriers and opportunities

1. What is your professional experience of mental healthcare in Cambodia?

2. In your experience with developing mental health services in Cambodia - what has been successful and what has not been successful and why?

3. How can the currently available mental health resources (e.g. staff, funding) in Cambodia be used in a better way? What are the barriers for doing so?

4. How can Cambodia ensure that its mental health work force has the adequate clinical knowledge and skills needed for appropriate clinical care? What are the barriers to achieving this?

5. How can effective mental health leadership be achieved in Cambodia? What are the barriers to achieving such leadership?

6. What types of mental health care services do you think should Cambodia invest in?

7. Do you have any further thoughts or comments on the barriers to developing mental healthcare in Cambodia or other ideas how it could be improved?

Please note: additional sub-questions may be used during the interviews if necessary to explain the question or to allow further elaboration of the interviewees answers.
