# Supplementary material for: Development of mental healthcare in Cambodia: barriers and opportunities
Source: Int J Ment Health Syst. 2020 Jul 29;14:53. doi: 10.1186/s13033-020-00385-4 (PMC7392648; doi:10.1186/s13033-020-00385-4)
Supplement: Supplementary file 2 — Additional file 2. Questions used in interview (Khmer). [file 13033_2020_385_MOESM2_ESM.pdf]

OMF International
#3, street 604
Tuol kork, Phnom Penh
Web: www.omf.org

ចំណងជើងការសិក្សាស្រាវជ្រាវ: ការអភិវឌ្ឍ ការថែទាំសុខភាពផ្លូវចិត្ត នៅកម្ពុជា: ឧបសគ្គ និងឱកាស

១. តើបទពិសោធន៍វិជ្ជាជីវៈ ផ្នែកសុខភាពផ្លូវចិត្តរបស់អ្នក នៅក្នុងប្រទេសកម្ពុជា ជាអ្វីដែរ?

២. បើផ្តោតទៅលើបទពិសោធន៏របស់អ្នក ក្នុងការអភិវឌ្ឍសេវាសុខភាពផ្លូវចិត្តនៅកម្ពុជា តើមានអ្វីខ្លះដែលទទួលបានជោគជ័យ? និងអ្វីខ្លះដែលមិនទទួលបានជោគជ័យ? សូមជួយប្រាប់អំពីមូលហេតុ?

៣. តើធនធានដែលមានស្រាប់ផ្នែកសុខភាពផ្លូវចិត្ត (ឧ. ធនធានមនុស្ស ឬមូលនិធិ) នៅក្នុងប្រទេសកម្ពុជាបច្ចុប្បន្ននេះ គួរធ្វើ ឬប្រើប្រាស់បែបណា ដើម្បីអោយកាន់តែប្រសើរឡើងថែមទៀត? ។ តើមានអ្វីខ្លះ ដែលជាឧបសគ្គសម្រាប់ការធ្វើដូច្នេះ?

៤. តើកម្ពុជាអាចធានាថា កម្លាំងការងារផ្នែកសុខភាពផ្លូវចិត្តរបស់ខ្លួន មានចំណេះដឹងនិងជំនាញព្យាបាលតាមតម្រូវការចាំបាច់ ដើម្បីផ្តល់ការថែទាំព្យាបាល អោយបានសមស្រប ដោយរបៀបណាដែរ? តើមានឧបសគ្គអ្វីខ្លះដែលអាចរារាំង មិនអោយការងារនេះអាចសម្រេចទៅបាន?

៥. តើការដឹកនាំក្នុងវិស័យសុខភាពផ្លូវចិត្តប្រកបដោយប្រសិទ្ធិភាព អាចនឹងសម្រេចបានតាមគោលដៅនៅកម្ពុជាដោយរបៀបណាដែរ? តើមានឧបសគ្គអ្វីខ្លះ ដែលអាចរារាំង មិនអោយការដឹកនាំបែបនេះ អាចសម្រេចទៅបាន?

៦. តើសេវាថែទាំសុខភាពផ្លូវចិត្តប្រភេទណាខ្លះ ដែលអ្នកគិតថាប្រទេសកម្ពុជាគួរតែ ធ្វើវិនិយោគ?

៧. តើអ្នកមានយោបល់ឬមតិអ្វីបន្ថែមទៀត ផ្តោតលើឧបសគ្គផ្សេងៗទៀតក្នុងការអភិវឌ្ឍន៍ ការថែទាំសុខភាពផ្លូវចិត្តនៅកម្ពុជា ឬអាចជាគំនិតយោបល់ផ្សេងទៀត ថាតើវាគួរតែកែលម្អអោយប្រសើរជាងនេះដោយរបៀបណា?

OMF International
#3, street 604
Tuol kork, Phnom Penh
Web: www.omf.org

កំណត់ចំណាំ: សំណួរតិចតួចបន្ថែមមួយចំនួន អាចត្រូវបានប្រើក្នុងកំឡុងពេលសំភាសន៍ប្រសិនបើចាំបាច់ ដើម្បីពន្យល់ពីសំនួរ ឬដើម្បីឱ្យអ្នកឆ្លើយសំណួរ ឆ្លើយតបបន្ថែម។
